# Sensors in the Detection of Abused Substances in Forensic Contexts: A Comprehensive Review

**DOI:** 10.3390/mi14122249

**Published:** 2023-12-17

**Authors:** Luana M. Rosendo, Mónica Antunes, Ana Y. Simão, Ana Teresa Brinca, Gonçalo Catarro, Rodrigo Pelixo, João Martinho, Bruno Pires, Sofia Soares, José Francisco Cascalheira, Luís Passarinha, Tiago Rosado, Mário Barroso, Eugenia Gallardo

**Affiliations:** 1 Centro de Investigação em Ciências da Saúde, Universidade da Beira Interior (CICS-UBI), 6200-506 Covilhã, Portugal; luanamay.rosendo@hotmail.com (L.M.R.); antunes.ss.monica@gmail.com (M.A.); anaaysa95@gmail.com (A.Y.S.); anabrinca99@gmail.com (A.T.B.); goncalo.catarro@hotmail.com (G.C.); pelixo.silva@ubi.pt (R.P.); joao.pedro.martinho@ubi.pt (J.M.); bruno.pinheiro.pires@ubi.pt (B.P.); sofia_soares_26@hotmail.com (S.S.); jfcascalheira@ubi.pt (J.F.C.); lpassarinha@fcsaude.ubi.pt (L.P.); tiago.rosado@ubi.pt (T.R.); 2 Laboratório de Fármaco-Toxicologia, UBIMedical, Universidade da Beira Interior, EM506, 6200-000 Covilhã, Portugal; 3 Departamento de Química, Universidade da Beira Interior, 6200-001 Covilhã, Portugal; 4 Associate Laboratory i4HB-Institute for Health and Bioeconomy, NOVA School of Science and Technology, Universidade NOVA de Lisboa, 2819-516 Caparica, Portugal; 5 UCIBIO-Applied Molecular Biosciences Unit, Chemistry Department, NOVA School of Science and Technology, 2829-516 Caparica, Portugal; 6 Centro Académico Clínico das Beiras (CACB)—Grupo de Problemas Relacionados com Toxicofilias, 6200-000 Covilhã, Portugal; 7 Serviço de Química e Toxicologia Forenses, Instituto de Medicina Legal e Ciências Forenses—Delegação do Sul, 1169-201 Lisboa, Portugal

**Keywords:** sensors, detection, drug of abuse, blood and derivatives, urine, oral fluid, sweat, exhaled breath, hair, solid samples

## Abstract

Forensic toxicology plays a pivotal role in elucidating the presence of drugs of abuse in both biological and solid samples, thereby aiding criminal investigations and public health initiatives. This review article explores the significance of sensor technologies in this field, focusing on diverse applications and their impact on the determination of drug abuse markers. This manuscript intends to review the transformative role of portable sensor technologies in detecting drugs of abuse in various samples. They offer precise, efficient, and real-time detection capabilities in both biological samples and solid substances. These sensors have become indispensable tools, with particular applications in various scenarios, including traffic stops, crime scenes, and workplace drug testing. The integration of portable sensor technologies in forensic toxicology is a remarkable advancement in the field. It has not only improved the speed and accuracy of drug abuse detection but has also extended the reach of forensic toxicology, making it more accessible and versatile. These advancements continue to shape forensic toxicology, ensuring swift, precise, and reliable results in criminal investigations and public health endeavours.

## 1. Introduction

The worldwide consequences of illicit drug use are remarkable and impact our society in several different ways. From the economic standpoint to the social and public health and safety concerns, drugs of abuse have been emerging rapidly in every corner of the world. As we delve into the intricate sphere of drugs of abuse, it becomes evident that this issue transcends geographical boundaries, affecting communities worldwide. The European Monitoring Centre for Drugs and Drug Addiction (EMCDDA) states that currently, illicit drugs are everywhere; everything with psychoactive properties can be used as an illicit drug; and everyone can be affected by this problem—either directly or indirectly [1].

On a global scale, the number of drug users is on the rise. The United Nations Office on Drugs and Crime (UNODC) reported that in 2020, 1 in every 17 people between the ages of 15 and 64 consumed these substances, achieving an alarming number of 5.8 per cent of the global population in this age group—a 23 per cent increase in the past ten years. Cannabis is still the most widely used substance, followed by amphetamines and cocaine, but opioids are responsible for a significant portion of drug-related deaths globally [2].

Europe continues to witness a diverse and dynamic drug scenery, as several different illicit drugs are widely available at high potency or purity. The problem of new emerging drugs such as new synthetic substances has also been particularly alarming in Europe—not only are their effects now known, but they are also frequently present in combination with common drugs. Users are increasingly and unknowingly consuming drugs that are combinations of different substances [1]. This translates to an increase in accidental overdoses and deaths, and is a new challenge for medical and toxicology teams.

The phenomenon of polydrug consumption is a recent challenge for forensic toxicology laboratories [1,2], which play a key role in public health strategies and policies. As new and more diverse new drugs appear on the market, in different forms, concentrations and preparations, the field must constantly be on track, which demands constant research by forensic toxicologists. 

Current drug detection methods rely on qualitative and quantitative analyses of biological material such as urine, blood, saliva, hair, breath, and sweat. Although these strategies can be useful in some situations, they have numerous methodological drawbacks [3].

Biosensors have emerged as vital instruments at the forefront of scientific and technical breakthroughs in the search for new answers to today’s critical concerns. These incredible instruments provide a window into the molecular world, allowing us to identify and measure certain biological or chemical molecules with extraordinary precision and speed, allowing for in situ detection [4]. Various sensor types, including electrochemical, optical, and colorimetric sensors, play a pivotal role in detecting drugs in biological samples, each offering distinct advantages and facing specific challenges [3,5,6].

**Figure 1:**
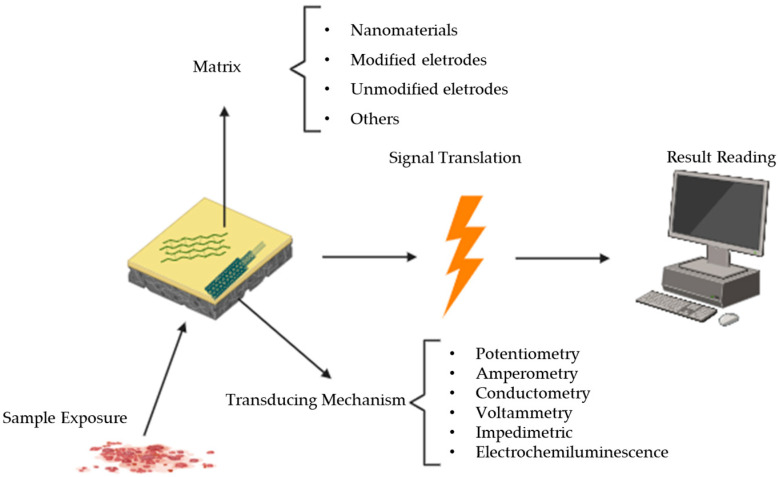
Illustration of electrochemical sensors and transduction principles.

Electrochemical biosensors, which harness the power of electrical impulses, offer unrivalled sensitivity and specificity. Because of their capacity to respond quickly to biochemical interactions at the electrode–solution interface, they are ideal for real-time monitoring, point-of-care testing, emergency situations, and quantitative analysis since the electrical signal generated is directly proportional to drug concentrations. Electrochemical biosensors are making an unmistakable impact across a wide range of fields, from clinical diagnostics to environmental monitoring and drug research. Electrochemical sensors operate by measuring changes in electrical properties, such as voltage or current, in response to the presence of drugs. The working principle involves the electrochemical reaction between the target substance and the sensor electrode, leading to measurable changes (Figure 1). For instance, voltammetric sensors employ cyclic voltammetry or differential pulse voltammetry for the detection and quantification of abused drugs like opioids. Amperometric sensors are suitable for drugs such as cocaine and cannabinoids, whereas potentiometric sensors excel in detecting ions or charged drug molecules. These sensors, including voltammetric and amperometric sensors, exhibit high sensitivity and specificity, particularly for substances with electroactive properties. Electrochemical sensors hold a prominent position in analytical applications due to their inherent advantages. Their operational versatility allows for real-time monitoring, making them well-suited for dynamic systems. These sensors are also amenable to miniaturization, facilitating integration into portable devices for on-site analyses. However, challenges persist, including the need for meticulous calibration to maintain accuracy, susceptibility to interference from complex sample matrices, and the potential for drift over time. Additionally, modifications with selective receptors are often necessary to enhance specificity for particular analytes, adding a layer of complexity to their deployment [3,7,8,9].

**Figure 2:**
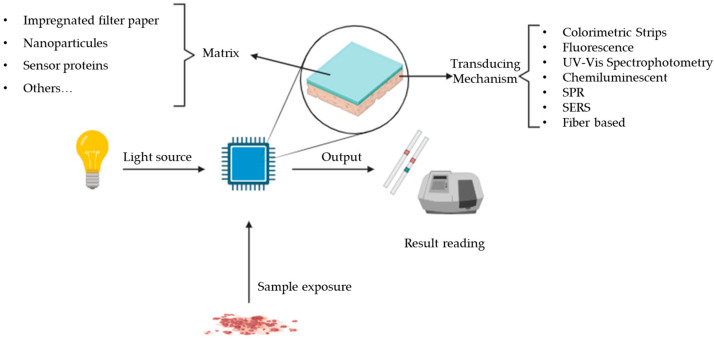
Optical sensors and transducing mechanism.

Optical biosensors, on the other hand, use changes in optical characteristics, such as absorbance, fluorescence, or refractive matrix, due to biochemical interactions between the recognition element and the target molecule. These types of biosensors excel in high sensitivity, multiplexing capabilities, and label-free detection. In the realms of biomedical research, drug discovery, environmental surveillance, and food safety, these devices offer insights that were once beyond our reach, and drug detection is no exception [3,5,6]. Optical sensors, including UV–Vis and fluorescence spectroscopy, utilize interactions between light and drug molecules for detection (Figure 2). These sensors offer high sensitivity and versatility. UV–Vis spectrophotometry measures light absorption, while fluorescence spectroscopy detects emitted light upon excitation. Optical sensors are advantageous for their non-invasiveness, but they may face challenges in complex sample matrices. Optical sensors, leveraging interactions between light and matter, offer distinctive advantages in analytical sensing. Their high sensitivity allows for the detection of low concentrations of analytes, and they often provide rapid and non-destructive measurements. Optical sensors are versatile and applicable across various sample matrices. Surface Plasmon Resonance (SPR) sensors, a subset of optical sensors, provide real-time insights into molecular interactions [10]. However, challenges include susceptibility to environmental conditions such as humidity and temperature, potential interference from complex sample matrices, and the necessity for careful calibration. Additionally, certain optical techniques, while powerful, may require sophisticated instrumentation, impacting their accessibility in certain settings [3,8,9].

Colorimetric sensors rely on visual colour changes as a result of a chemical reaction with the target substance. These sensors are simple, cost-effective, and often provide rapid results. They are advantageous for their ease of use and straightforward interpretation. However, colorimetric assays may lack the sensitivity and specificity exhibited by electrochemical and optical sensors. Nevertheless, challenges include the potential for interference from co-existing electroactive species, the requirement for precise control of experimental conditions to maintain accuracy, and susceptibility to contamination, which can impact their reliability. Additionally, these sensors may need sophisticated instrumentation, contributing to considerations of cost and accessibility in certain applications [11].

The choice of sensor type for drug detection in biological samples depends on factors such as the specific drugs of interest (selectivity), required sensitivity, and the nature of the sample matrix. Selectivity is the most important feature of the sensor since it demands meticulous consideration due to its direct impact on the reliability and precision of drug detection. Achieving selectivity involves the sensor’s ability to discern and respond exclusively to the target analyte amidst a complex sample matrix. This complexity often encompasses numerous interferences, including endogenous biomolecules, environmental contaminants, and structurally similar compounds [9,12]. One approach to enhancing selectivity is the integration of molecular recognition elements, such as aptamers, antibodies, or molecularly imprinted polymers, into the sensor design. These elements impart specificity by selectively binding to the target analyte, minimising cross-reactivity with interfering substances. Advancements in nanotechnology contribute to the development of nanomaterial-based selectivity strategies, leveraging unique properties to enhance discrimination between analytes [12,13].

Electrochemical sensors offer high precision, optical sensors provide versatility, and colorimetric sensors offer simplicity. Integrating these technologies allows for comprehensive and reliable drug detection in forensic and clinical applications. In the context of drug detection, this capability is crucial for monitoring drug levels in biological tissues, pharmaceutical manufacturing, and even law enforcement agencies to detect illicit drugs on-site [3].

This paper significantly contributes to the field of forensic science by providing a thorough review of recent advancements and varied applications of cutting-edge technologies for the analysis and detection of drugs of abuse. It stands out from the existing literature by focusing exclusively on publications from 2020 to the present, showcasing the latest developments in early drug detection across conventional and alternative biological matrices, as well as solid samples. As of now, no such comprehensive review dedicated to this topic has been published. The criteria for article selection and inclusion are presented in the Supplementary Information. The inclusion of this latter section enhances the reliability and relevance of the findings, offering additional insights into the contemporary research and methodologies employed in the last few years.

## 2. Classification

This review article explores the significance of biological and seized drug samples in forensic toxicology and highlights the transformative impact of portable sensors in enhancing precision, efficiency, and portability of sample analysis.

Biological matrices such as blood, urine, oral fluid, sweat, and hair provide important information concerning human exposure, and each of these matrices offers unique advantages (Table 1). Blood and derivatives provide real-time data on drug levels, offering a snapshot of immediate exposure and potential intoxication. Urine, with its non-invasive and easy-to-collect nature, affords a broader time window for detecting drug metabolites, chronic drug use, and patterns of consumption. Oral fluid and sweat, though less commonly explored, offer valuable insights into recent drug use. Hair, on the other hand, provides a historical record of drug use over months. It has proven instrumental in identifying chronic drug abusers, providing a unique perspective [6,14,15].

In parallel, the analysis of solid samples like seized drugs and powders may aid law enforcement agencies and forensic scientists in drug identification, profiling, and tracking the sources of illicit substances. Solid samples enable forensic toxicologists to determine the purity of seized substances, contributing to public health initiatives aimed at mitigating the risks associated with adulterated drugs. Furthermore, the identification of novel synthetic compounds and emerging substances in solid samples allows forensic scientists to keep pace with the ever-evolving landscape of designer drugs. These samples are vital not only for uncovering trafficking routes but also for elucidating the potency and purity of street drugs, shedding light on potential public health hazards.

In recent years, forensic toxicology has undergone a transformative shift with the integration of portable sensor technologies, including spectroscopy, chromatography, and electrochemistry. These devices have significantly enhanced the analysis of biological and solid samples. Their portability allows for on-site and near-site testing, resulting in reduced turnaround times, preserved sample integrity, and heightened investigative efficiency, particularly in cases of suspected intoxication or substance identification. Portable sensors have become vital tools for rapid screening of biological samples in various scenarios, such as traffic stops, crime scenes, and workplace drug testing, enabling the swift detection of a wide range of substances from alcohol and illicit drugs to toxic chemicals, all within minutes.

In the subsequent section, we present a comprehensive review of the diverse applications of various types of sensors for detecting drugs of abuse in biological and solid samples.

### 2.1. Blood and Derivatives

Developing accurate drug detection in the complex blood matrix, encompassing plasma and serum, is challenging due to matrix effects from drug interactions with blood components. These effects can impair sensor performance, demanding research to understand and mitigate them for enhanced drug detection reliability in plasma and serum. Forensic and clinical applications require sensitivity and specificity to detect drugs at low concentrations, emphasising research directions aimed at improving sensor sensitivity and specificity for reliable identification of trace substances. Biological variability in plasma and serum samples, arising from individual differences in blood composition and drug metabolism, poses challenges for consistent sensor performance, prompting research into strategies addressing this variability. Real-time monitoring of dynamic changes in drug concentrations is crucial, leading to the development of sensors providing timely and accurate information for forensic investigations and medical diagnostics. Multiplexed detection, enabling simultaneous identification of multiple drugs, shows promise for enhancing drug screening efficiency. Long-term stability of sensors in these samples needs study on materials and designs ensuring sustained performance. Additionally, understanding and mitigating interference from various substances in plasma and serum constitute key research areas for improving drug detection specificity [3,16].

Table 2 summarizes various methods employed for the detection of abused drugs in blood and derivatives, including plasma and serum.

For instance, different methodologies for the detection of methamphetamine (MA) are described. The first method employs an optical sensor based on a portable chemiluminescent fiber-based immunosensor (PCFS). This sensor is highly sensitive, boasting a low detection limit of 0.5 ng/mL in blood samples [17]. In contrast, the second method utilizes an electrochemical aptamer-based (EAB) sensor with a detection limit of 30 nM in serum. A notable distinction is that the second method does not require the use of reagents, offering significantly higher sensitivity in MA detection [18].

Other applications for these types of samples involve two distinct approaches for the determination of methadone (MET), employing electrochemical sensors. The first sensor requires the modification of a glassy carbon electrode with a thin layer of poly L-arginine (P-L-Arg/GCE), achieved through the electropolymerisation of L-arginine monomers using cyclic voltammetry (CV). This sensor, when optimised, exhibits a linear response to MET concentrations within the ranges of 0.49–2.98 µM and 2.98–11.9 µM, with a detection limit of 0.32 µM. It has successfully been employed for serum samples, demonstrating its potential for real sample analysis with acceptable recovery rates [19]. The second sensor features a glassy carbon electrode modified with two layers of a graphene/Ag nanoparticles (Gr/AgNPs) nanocomposite (Gr/AgNPs)2/GCE). This sensor showcases remarkable sensitivity to MET within the concentration range of 1.0–200.0 µM, boasting an impressive detection limit of 0.12 µM. Furthermore, this electrode exhibits strong selectivity for MET, rendering it suitable for the analysis of real samples such as human blood serum [20].

### 2.2. Urine

Table 3 presents various types of sensors employed to ascertain the presence of different illicit drugs in urine samples. Over recent years, there has been a notable diversification in the methods employed, ranging from optical to electrochemical sensors. For instance, optical sensors have been used in the detection of substances such as fentanyl, ketamine, morphine, GHB, methcathinone, methamphetamine, and MDMA. On the other hand, electrochemical sensors have also proven effective in detecting ketamine, fentanyl, MDMA, amphetamine, methamphetamine, methadone, tramadol, and diazepam.

In the literature, various methods report using different sensors to detect the same substance. For example, in the case of ketamine, the first method described entails the development of an electrochemical aptamer-based (EAB) sensor. This sensor exhibits notable attributes, including rapidity, sensitivity, and solventless operation, yielding nanomolar detection accuracy. Results are obtainable within 30 s. This sensor detects ketamine in undiluted urine, achieving detection limits as low as 10 nM, significantly surpassing the physiological detection threshold [27]. The second method introduces a pioneering surface plasmon resonance (SPR)-based gene–nanoparticle system tailored for the detection of various narcotic drugs. The primary objective is to establish an optical sensor array. It is possible to observe distinct differences in the UV–Vis spectra of DNA–gold nanoparticles in the presence of these drugs. To ensure accurate classification, partial least squares discriminant analysis (PLS-DA) is employed, successfully categorising these drugs in human urine. The integration of a multi-sensor unit further enhances the predictive accuracy of the PLS-DA models. This innovative approach holds considerable potential for on-site drug detection and screening for drug abuse [28].

Regarding the presence of fentanyl in urine samples, a novel enzymatic biosensor relies on a cost-effective and reliable detection for this compound. This innovative sensor employs disposable screen-printed carbon electrodes modified with multi-walled carbon nanotubes and cytochrome c, offering a simple method with a wide detection range, high sensitivity, and low detection limits (0.086 μg/mL) [29]. On the other hand, a visual colorimetric assay is also discussed in Table 2, showcasing its effectiveness in diverse settings. This assay achieves a low detection limit (0.9 mg/L) in urine samples within a short time frame (6 min). Importantly, it demonstrates resilience against interference from various substances, including opioids and contaminants commonly found in street drugs. Additionally, a smartphone-based portable device for fentanyl detection was successfully developed, suitable for on-site field tests [30].

Lastly, MDA and MDMA received vast attention due to their widespread use, and law enforcement organizations are required to identify them on-site in unknown samples quickly and reliably. As shown in Table 2, an electrochemical sensor based on molecularly imprinted polydopamine (MIP@PDA) was designed to detect these illicit compounds by differential pulse voltammetry (DPV). Because of the greater affinity of MIP@PDA to the target, it supplied many binding sites and expanded the practical application of the sensor. The sensor demonstrated outstanding analytical performance, with LODs of 37 nM and 54 nM for MDA and MDMA, respectively. Furthermore, this sensor demonstrated suitable selectivity, stability, repeatability, and detection ability in urine samples, indicating a good choice for a quick diagnostic approach in drug investigations [31]. Still regarding the study of the two amphetamine-type stimulants in urine samples, an efficient electrochemical-surface plasmon resonance (EC-SPR) sensor was also demonstrated, coupled with a molecularly imprinted strategy, for adsorption and quantitative measurement. This method presented a lower detection limit with 57 nM and 59 nM for MDA and MDMA in broad linearity, and it could resist the interferences from assorted substances. Furthermore, the EC-SPR sensor detected spiked MDMA in urine samples, indicating considerable application possibilities in forensic investigation [22].

### 2.3. Oral Fluid

Oral fluid is considered an alternative matrix when compared to classical biological samples, being one of the easiest specimens to implement in sensor systems for drug detection. This biological specimen has been applied to the detection of several drugs of abuse, and Table 4 summarizes the sensor procedures developed and published from 2020 to the present year in which oral fluid is analysed.

Methamphetamine is the most detected drug of abuse by these sensor systems in oral fluid samples. With the implementation of electrochemical sensors with different transduction mechanisms, Dokuzparmak et al. [23] were able to obtain a LOD of 10 µM, while Xie et al. [18] obtained a LOD value in the order of 20 nM. However, Ghorbanizamani et al. [39] achieved a significantly lower LOD value of 0.72 ng/mL. On the other hand, and with the use of optical sensors, Yao et al. [40] obtained a LOD value of 0.95 ng/mL for methamphetamine with the application of 1 mL of biological sample, and Zhao et al. [17] were able to achieve a LOD of 0.5 ng/mL for the same drug and with just 10 min of analysis, values similar to the work mentioned previously of Ghorbanizamani et al. [39], for an electrochemical sensor. In the case of amphetamine, and as for methamphetamine, Dokuzparmak et al. [23] achieved a LOD value of 10 µM, while Beduk et al. [41] obtained a substantially lower value of 9.7 ng/mL for the detection of the same drug of abuse.

Another example is cocaine, for which Parrilla et al. [42] obtained a LOD of 1.2 µM with the implementation of an electrochemical sensor. More recently, and for the same type of sensor, Beduk et al. [41] obtained a lower LOD of 4.3 ng/mL. However, with the use of 1 mL of biological specimen and an optical sensor by Yao et al. [34], and the implementation of a colorimetric sensor with an analysis time of less than 90 min by Sanli et al. [43], LOD values significantly lower than those previously described were obtained of 3.14 ng/mL and 0.97 nM, respectively.

Regarding ketamine, Parrilla et al. [42] also managed to detect it in a multimethod capable of identifying four drugs of abuse, at a LOD of 2.6 µM. On the other hand, and also with the implementation of an electrochemical sensor, Xie et al. [27] developed a methodology for detecting ketamine in just 30 s, for which it was possible to obtain a LOD value of 10 nM.

It should also be noted that, for the detection of synthetic cannabinoids with the development of an electrochemical-type sensor, Akgönüllü et al. [44] reached LODs and limits of quantification values in the order of pg/mL, between 0.23 to 0.3 pg/mL and 2.4 to 3.1 pg/mL, respectively.

In general, all the authors of these works concluded that the information obtained through these sensors and developed methodologies is extremely relevant in workplace and roadside environments, for example, and as a way of streamlining and facilitating processes and complementing analytical methodologies such as gas or liquid chromatography.

### 2.4. Sweat

Sweat is an alternative biological matrix that also has the potential to be analysed by these systems. According to the literature, only two works were published in which this biological sample is used for the detection of drugs of abuse with sensor systems.

The first work (Table 5) describes the development of a wearable surface-enhanced Raman scattering (SERS) sensor as a type of patch to be used as a molecular sweat sensor. The feasibility of its use as an optical sensor was demonstrated by the detection of 2-fluoro-methamphetamine, an analogue of methamphetamine, through application of spiked-simulated sweat in human cadaver skin. The time of this detection is affected by factors such as sweat amount, humidity, and temperature of the environment; the limit is not lower than that of the method analysis by gas chromatography coupled to mass spectrometry, and it is necessary to use the patch for a longer period of time. However, detection is performed without detaching the patch from the skin. The authors believe that the developed SERS patch sensor can be used as various flexible and wearable biosensors for health monitoring, with further modification of the silver nanowires with bio/chemical receptors [48]. The second publication, by Zhang et al. [49], proposes a wearable electrochemical aptasensor for rapid detection of several drugs in sweat, such as cathinones, cocaine, and heroin. Aptamers with different compositions were developed for different binding affinities and proved to be sufficient to generate distinct electrochemical fingerprints for different psychoactive drugs and interfering substances, with thirteen drugs being identified in the same concentration or gradient concentrations. As an example, cathinone presented a LOD that varies between 0.18 and 0.57 nM, depending on the aptamer or set of aptamers implemented. Furthermore, the applicability of the sensor in distinguishing drugs with similar chemical structures in samples of artificial sweat and human sweat was demonstrated. The authors concluded that the sensor array provides a new easy, fast, and reliable method for detecting psychoactive drugs and serves as a reference for the development of sensors for daily testing of human biochemical information.

### 2.5. Hair

Hair is another alternative matrix that is currently used in forensic testing due to its high stability, easy sample collection, and ability to hold drugs of abuse for several months after consumption. Although the usage of this matrix is mostly to obtain a back log of previously consumed drugs dating several months prior to its collection, it may also present a significant potential when it comes to its application in drug testing sensors (Table 6).

The studies by Guo et al. [50] and Fan et al. [51] were the only articles found in this search in which sensors were created for the detection of drugs in human hair samples. Both authors utilized optical sensors for this detection, with Fan et al. [51] being able to detect methamphetamine and morphine and Guo et al. [50] ketamine.

Guo et al. [50] created a cloud-enabled smartphone-based fluorescence sensor based on the ketamine antibody–antigen (Ab-Ag) specific binding to influence the fluorescence of the sensor.

Fan et al. [51] took a slightly different route and created a deeply dyed nanobead matrix through oil/water mini emulsion-solvent evaporation techniques, widely used to prepare functional nanobeads by encapsulation, as a more sensitive alternative to traditional gold nanoparticle-based lateral flow immunoassay strips which allows for the detection of methamphetamine and morphine with the naked eye.

Comparing obtained LODs and times of analysis, Guo et al. [50] achieved a lower LOD (1 ng/mL) for ketamine with an analysis time around the 5 min mark. Fan et al. [51] did not stay far from those values, with an LOD of 8 ng/mL within 8 min. This might be due to the fact that Guo et al. [50] used a fluorescence-based mechanism, while Fan et al. [51] employed colorimetric strips.

It is safe to say that the sensors developed by these authors were not only fast but also allowed obtaining quite low LODs for the amount of hair used, with Fan et al. [51] having the extra advantage of the naked eye detection.

### 2.6. Exhaled Air

Exhaled air has been used for several years to detect blood alcohol concentrations in order to control drunk drivers, prevent accidents, and improve road safety overall. This matrix is easy to obtain in the moment, without presenting any invasive procedures towards the individual, and holds a high potential when it comes to sensor development.

Concerning this sample, only four articles were identified in this search (Table 7). The work by Biswas and Saha [52] stands out notably, primarily due to the unconventional sensor they employed. These authors devised and assessed an electrical sensor prototype, departing from the conventional chemical and optical sensors typically utilized [52]. In their study, they utilized a metal oxide semiconductor (MOS) known as MQ3, composed of SnO_2_, which exhibits variations in conductivity when exposed to alcohol [52]. This property enables the detection of alcohol within a working range spanning approximately from 230–800 ng/mL almost instantly (8 s) [52].

In essence, their approach hinges on a straightforward principle: SnO_2_ demonstrates reduced conductivity in the presence of clean air, and its conductivity rises proportionally with alcohol concentration [52]. Owing to this mode of detection, the MQ3 alcohol sensor can produce both digital and analogue outputs, rendering it readily integrable with microcontrollers and Arduino boards [52].

Both studies, Gusso et al. [53] and Hwang et al. [54], employed chemical sensors based on single-walled carbon nanotubes (SWCNT) as substrate. SWCNTs offer exceptional suitability for the detection of minute concentrations of both chemical and biological compounds, attributable to their extensive surface area and responsiveness measured through changes in electrical resistance.

While Gusso et al. [53] adhered to the traditional path of ethanol (EtOH) detection, Hwang et al. [54] took a distinctive approach by applying this sensor concept to the detection of tetrahydrocannabinol (THC). Achieving selectivity for THC vapour amidst other more volatile compounds present in breath, such as CO_2_ and water, was accomplished by implementing a delay in the sensor’s readout [54]. This delay allowed for the desorption of these interfering compounds from the sensor’s surface [54]. Consequently, this influenced the duration of the analysis [54].

In comparison to the rapid 5 s analysis achieved Gusso et al. [53], the extended 15 min analysis time in Hwang et al. [54] appears considerably longer. However, this extended duration remains justifiable, primarily due to the necessity of ensuring selectivity towards THC [54].

In Mustafa et al. [55], colorimetric strips were employed within an optical sensor configuration, utilizing Cerium nanoparticles (CeNPs) as the matrix in conjunction with the enzyme alcohol oxidase (ALOx). This combination served for the collection, analysis, and generation of a signal that can be discerned with the naked eye [55]. This compact and portable device exhibits sensitivity and selectivity of such calibre that it can detect ethanol (EtOH) concentrations at a level on par with electronic breathalysers [55].

The detection mechanism employed is relatively straightforward: when ethanol and ALOx are combined, ethanol undergoes enzymatic oxidation to form acetaldehyde, concurrently producing hydrogen peroxide (H_2_O_2_) as a byproduct [55]. This resulting byproduct then reacts with the CeNPs, leading to a discernible change in colour that is directly proportional to the concentration of H_2_O_2_, and, consequently, the EtOH concentration [55]. This visible colour transformation initiates immediately upon contact, reaching its peak and stabilizing after approximately 15 min [55].

The LODs achieved in these studies appears to be consistently low. However, it is challenging to make direct comparisons between them due to the lack of standardised information and variability in measurement units employed.

### 2.7. Vapours

Vapours function as an intriguing matrix for drug-detecting sensors. These sensors are capable of selectively detecting and identifying drugs or illicit substances when they are volatilized and released into the air as vapours, enabling rapid and non-invasive drug detection (Table 8).

Cali and Persaud [56] adopted a novel and distinctive approach for the development of a sensor designed to detect drugs of abuse. The authors ingeniously crafted mutant odorant-binding proteins (OBP) derived from insects, specifically Anopheles gambiae. These mutant OBPs demonstrated the remarkable ability to bind to the analytes of interest, serving as a potential matrix for a fluorescence sensor [56].

To facilitate this detection, immobilization of these OBPs on Quartz Crystal Microbalances (QCMs) was performed [56]. The outcomes were particularly noteworthy, as these immobilized OBPs exhibited a high degree of affinity for several key substances, including cannabinol (CBD), MDMA, and cocaine [56].

Li et al. [57] and Liu et al. [58] adopted optical approaches in their quest to develop sensors for drug detection. In Li et al. [57], the chosen analyte was methylphenethylamine (MPEA), a stimulant drug similar to methamphetamine. Conversely, in Liu et al. [58], a broader spectrum of drugs, including methamphetamine, MDMA, ketamine, magu, and phenobarbital, were targeted for detection.

Li et al. [57] designed a fluorescent polymer probe, denoted as S1. This probe featured a conjugated polymer with a conjugated thiophene ring structure [57]. When coupled with a straightforward and efficient ultraviolet (UV)–ozone substrate treatment, it exhibited the ability to detect the drug of interest with more sensitivity. The LOD achieved was impressively low, with values as low as 2.59 ng/mL for the S1a matrix film and a mere 0.25 ng/mL for S1d [57].

Liu et al. [58] took a different approach by employing perylene bisimide derivatives (PBIs), known for their exceptional fluorescence properties when detecting organics containing amido bonds. In this study, an o-carborane derivative of PBIs, termed PBI-CB, demonstrated significant sensing capabilities for the vapours of various illicit drugs [58]. Additionally, Liu et al. [58] pioneered the development of a prototype fluorescence sensor comprising two films, which enabled quicker sensing tests with reduced energy consumption.

While the time required for analysis in Li et al. [57] is more than double that of Liu et al. [58], both sensors demonstrate remarkable speed when it comes to drug detection.

Zhang et al. [59] and Liang et al. [60] adopted a chemical approach, focusing on sensors based on conductivity. In Zhang et al. [59], a P3CT polymer (poly[3-(6-carboxyhexyl)thiophene-2,5-diyl]) was functionalised with carbon nanotubes to detect methamphetamine. Remarkably, this sensor achieved a very low LOD, at just 4 µg/L [59]. To validate the sensor’s effectiveness, the authors utilized *N*-methylphenethylamine (NMPEA) as a test analyte due to its structural similarity to *N*-methamphetamine (NMPA) [59]. Overall, this approach yielded a rapid and substantial signal, while the sensor maintained semi-recoverable characteristics [59].

In Liang et al. [60], the authors pursued an easily and rapidly synthesized sensor utilizing rhombohedral In2O3 nanoparticles, employing a microwave-assisted hydrothermal pathway. This sensor was tailored for the detection of EtOH and demonstrated a significantly higher response compared to other sensors employing m-In2O3 and c-In2O3 [60].The LOD achieved in this case was approximately 330 µg/L [60]. Notably, the time required for analysis in both was approximately the same.

### 2.8. Illicit Drugs in Solid Samples

According to the literature (Table 9), electrochemical sensors with SWV as a tracing mechanism are commonly used for solid samples such as powders, tablets, and seized drugs, among others. However, the sensor matrix varies depending on the specific type of abused drug under investigation and the required characteristics for achieving greater accuracy. For instance, this study [61] presents a rapid electrochemical detection method for amphetamines in seized samples using SWV at graphite screen-printed electrodes (SPEs). This method, involving derivatisation by 1,2-naphthoquinone-4-sulfonate (NQS), represents the first demonstrated for quantification in drug seizures. The sensor’s analytical performance demonstrates the feasibility of detecting and quantifying AMP in approximately 3 min for 20 confiscated samples, with a LOD of 22.2 µM [61]. In comparison, a paraformaldehyde-coated (PFA) sensor has been developed for the on-site detection of amphetamine in seized drug samples. This sensor, utilizing PFA coating on a graphite screen-printed electrode and PBS pH 12, can detect AMP in the presence of common diluents and adulterants, including caffeine. The qualitative analysis, fully developed and validated, requires only 1.5 min and has a LOD of 0.3 µM. The sensor’s affordability and rapid nature position it as a potential alternative for on-site analysis of various suspicious samples, particularly for detecting illicit drugs [62]. In summary, these sensors offer rapid, accurate, and sensitive methods for detecting and quantifying AMP in confiscated samples, highlighting their affordability and potential for on-site analysis of suspicious samples, particularly for detecting illicit drugs.

Another example for the analysis of the samples in question pertains to the development of two methods for determining 1-(3-chlorophenyl)piperazine (mCPP). The first method employs a cathodically pre-treated boron-doped diamond electrode (CPT-BDDE), yielding a LOD of 1.1 μM [63]. The second method utilizes disposable screen-printed carbon electrodes (SPCE) and rapid screening procedures with minimal sample sizes (100 μL), resulting in a LOD of 0.1 μM [64].

Voltammetric methods for analysing narcotics have gained popularity due to their portability and affordability. Paper substrates, graphite pencils, and silver paint can be used to construct paper-based electrodes. A paper-based device equipped with three electrodes was compared to a commercially available screen-printed carbon electrode. SWV was employed for the analysis of lysergic acid diethylamide (LSD), yielding detection and quantification limits of 0.38 and 1.27 μM, respectively. The paper-based electrodes and the operational methodologies developed were shown to be suitable for LSD detection and quantification, providing results similar to those obtained with commercial screen-printed electrodes and chromatographic analysis [65].

## 3. Future Perspectives

Future research on electrochemical and optical sensors for drug detection holds substantial promise, with a focus on advanced analytical methodologies. In the realm of electrochemical sensors, novel materials and designs are crucial for enhancing sensitivity, selectivity, and stability. The integration of nanotechnology and advanced signal processing techniques shows potential for high-performance sensors, although addressing challenges related to sample matrix complexities and sensor calibration is essential for practical implementation [8,9,13]. On the optical sensing front, opportunities for innovation include technologies like Surface Plasmon Resonance (SPR) and Raman spectroscopy, with an emphasis on improving robustness and portability for on-site and point-of-care applications [5,10]. Incorporating artificial intelligence (AI) and machine learning (ML) algorithms is a notable avenue for optimizing data analysis and enhancing sensor reliability in both electrochemical and optical sensors [9,13,69,70]. AI and ML are anticipated to play pivotal roles in revolutionizing biosensors [71]. These technologies can optimize sensor performance by analysing complex data patterns, improving accuracy, and enabling real-time adaptive adjustments [71]. ML, when coupled with electrochemical sensors, has the potential to contribute to more robust calibration methods, addressing challenges related to sample matrix complexities and achieving higher sensitivity [71].

Advancements in miniaturisation and integration with microfluidic systems offer potential for rapid, efficient, and real-time drug detection in both electrochemical and optical sensor types [72]. Exploring flexible and wearable sensor platforms for continuous monitoring is an exciting direction for research. The future of biosensors for drug detection hinges on transformative developments through cutting-edge technology integration, with AI and ML expected to play pivotal roles in improving sensor performance [3,6,9]. Since these technologies can optimize sensor performance by analysing complex data patterns, they may improve accuracy and enable real-time adaptive adjustments.

The integration of biosensors with the Internet of Things (IoT) presents a futuristic avenue for enhanced connectivity and data management, enabling real-time transmission of sensor data for analysis and immediate responses. Nanotechnology and advanced materials could revolutionize biosensor design, enhancing sensitivity and selectivity. Flexible and wearable biosensor platforms hold promise for various applications, including personalised healthcare and forensic investigations [73].

## 4. Conclusions

In conclusion, the field of forensic toxicology has witnessed significant advancements in sensor technologies, leading to improved precision and efficiency in the detection of drugs of abuse across various sample matrices. Our analysis has highlighted some key trends and areas for further exploration.

The electrochemical sensor stands out as the most widely used sensor type, demonstrating its versatility and effectiveness in detecting drugs of abuse across different sample matrices. This prevalence speaks to its reliability and suitability for a wide range of applications.

When it comes to biological samples, blood, urine, and saliva have been the focal points of sensor development, boasting a variety of sensor types tailored to their unique characteristics. This diversity in sensor applications underscores the importance of customising sensor technology for specific matrices to enhance detection capabilities (selectivity and sensitivity) and accuracy.

However, the landscape of hair and sweat analysis remains relatively unexplored, with only limited research available. These matrices hold promise for long-term drug exposure monitoring, and further research is imperative to unlock their full potential.

In the realm of solid samples, the concept of gloved sensors presents an intriguing future perspective. While this innovative approach holds promise for on-site detection of drug substances, it is crucial to emphasize the need for more research and practical application in the field. These gloved sensors have the potential to revolutionize drug detection in seized substances, but rigorous testing and validation are essential to ensure their reliability and accuracy.

The integration of AI, ML, IoT, and other modern technologies represents a dynamic frontier in the evolution of biosensors for drug detection. The synergy of these advancements is expected to propel biosensors towards unprecedented levels of accuracy, efficiency, and adaptability, opening new horizons for applications in diverse fields. Ongoing research accomplishments focus on addressing current limitations, advancing sensor technologies, and optimizing their practical utility, all while embracing the potential of these integrated technologies for enhancing sensing variability.

Overall, this review underscores the importance of sensor technologies in forensic toxicology and highlights the need for ongoing research and development to harness the full potential of these tools. As technology continues to advance, forensic toxicologists can expect even more precise and efficient methods for detecting drugs of abuse in various sample matrices, ultimately enhancing the field’s ability to contribute to criminal investigations and public health initiatives.

## Figures and Tables

**Table 1:** Summary of biological and seized drug samples in forensic context and their advantages.

Samples	Advantages	Forensic Significance
Blood and Derivatives	Rich genetic material; precise analysis	Accurate identification, drug detection, toxicological assessments
Urine	Non-invasive; accumulates metabolites	Drug screening, substance use monitoring, toxicology evaluations
Oral Fluid	Easily collected; reflects recent intake	Detects drugs, alcohol, and toxins; valuable in roadside testing
Sweat	Reflects recent exposure; continuous excretion	Identifies recent drug use, complements other sample analyses
Hair	Long-term history of substance exposure	Reveals chronic drug use, retrospective analysis of substance use
Exhaled Air	Non-invasive; real-time monitoring	Detects volatile substances, aids immediate intoxication assessment
Vapours	Captures volatile compounds in immediate vicinity	Identifies substances at crime scenes, aids in forensic analysis
Solid Samples	Diverse material sources (e.g., tablets, powder, seized samples); varied composition; convenient handling	Identifies and quantifies drugs, crucial for legal proceedings

**Table 2:** Detection systems for blood and derivatives (serum, plasma).

Compounds	Sample Volume	Type of Sensor	Transducing Mechanism	Sensor Matrix	LOD	LOQ	Time of Analysis	Ref.
Methamphetamine	n.s.	Optical	PCFS	n.s.	0.5 ng/mL	1.5 ng/mL	10 min	[17]
Methamphetamine	n.s.	Electrochemical	SWV	Apt-38-MB	30 nM	n.s	n.s	[18]
Methadone	2 mL	Electrochemical	DPV	P-L-Arg/GCE	0.032 μM	n.s	n.s	[19]
Methadone	1 mL	Electrochemical	DPV	(Gr/AgNPs)_2_/ GCE	0.12 μM	n.s	n.s	[20]
Tramadol	n.s.	Electrochemical	CV	1-M-3-BBr/Pr(OH)_3_-GQD/CPE	3.0 × 10^−9^ [M]	n.s.	n.s	[21]
(a) MDA (b) MDMA	n.s.	Optical	SPR	MIPs nanofilm	(a) 57 nM (b) 59 nM	n.s	n.s	[22]
Methamphetamine Amphetamine	n.s.	Electrochemical	Electrochemiluminescence	Nafion-[Ru(bpy)_3_]^2+^	0.2 μM	n.s.	n.s.	[23]
(a) Morphine (b) MDMA	3 mL	Electrochemical	DPV	CNHs-CHI@PtNPs/GCE	(a) 0.02 (b) 0.018	n.s.	n.s.	[24]
Diazepam	n.s.	Electrochemical	Potentiometry	Cu/POT/ISM	1.2 × 10^−7^ [M]	n.s.	11 ± 2 s	[25]
Oxycodone	n.s.	Electrochemical	DPV	Nafion/SWCNT	85 nM	n.s.	n.s.	[26]

1-M-3-BBr/Pr(OH)3-GQD/CPE: 1-methyl-3-butyl imidazolium bromide/praseodymium hydroxide-graphene quantum dots nanocomposite modified carbon paste electrode; Apt-38-MB: aptamer-38-methylene blue; CNHs-CHI@PtNPs/GCE: high surface area carbon nanohorns decorated with Pt nanoparticles; Cu/POT/ISM: copper-poly(3-octylthiophene)-ion-sensitive membrane; CV: cyclic voltammetry; DPV: differential pulse voltammetry; (Gr/AgNPs)_2_/GCE: glassy carbon electrode modified with two layers of graphene/Ag nanoparticles; LOD: limit of detection; LOQ: limit of quantification; MIP: molecular imprinted polymer; Nafion-[Ru(bpy)_3_]^2+^: Nafion and tris(2,2’-bipyridyl)-dichlororuthenium(II) hexahydrate; Nafion/SWCNT: nafion and single-walled carbon nanotube; n.s.: not specified; PCFS: portable chemiluminescent fiber-based immunosensor; P-L-Arg/GCE: glassy carbon electrode modified by a thin layer of poly L-arginine; SPR: surface plasmon resonance; SWV: square wave voltammetry.

**Table 3:** Detection systems for urine.

Compounds	Sample Volume	Type of Sensor	Transducing Mechanism	Sensor Matrix	LOD	LOQ	Time of Analysis	Ref.
Methamphetamine	n.s.	Optical	PCFS	n.s	0.5 ng/mL	1.5 ng/mL	10 min	[17]
Methamphetamine	n.s.	Electrochemical	SWV	Apt-38-MB	50 nM	n.s.	n.s.	[18]
Methadone	2 mL	Electrochemical	DPV	P-L-Arg/GCE	0.032 μM	n.s.	n.s.	[19]
(a) MDA (b) MDMA	n.s.	Optical	SPR	MIPs nanofilm	(a) 57 nM (b) 59 nM	n.s.	n.s.	[22]
MethamphetamineAmphetamine	n.s.	Electrochemical	Electrochemiluminescence	Nafion-[Ru(bpy)_3_]^2+^	0.2 μM	n.s.	n.s.	[23]
(a) MDMA (b) Morphine	3 mL	Electrochemical	DPV	CNHs-CHI@PtNPs/GCE	(a) 0.018 μmol/L (b) 0.02 μmol/L	n.s.	n.s.	[24]
Diazepam	n.s.	Electrochemical	POT	Cu/POT/ISM	1.2 × 10^−7^ [M]	n.s.	11 ± 2 s	[25]
Ketamine	n.s.	Electrochemical	EAB	Au/Apt/MCH	10 nM	n.s.	30 s	[27]
(a) Pentobarbitol (b) Caffeine (c) Morphine (d) Remifentanil (e) Fentanyl (f) Ketamine (g) Etomidate (h) Carfentanil (i) Sulfentanyl	n.s.	Optical	PLS-DA	AuNP	n.s.	n.s.	n.s.	[28]
Fentanyl	150 μL	Electrochemical	AdSCV	Cytc/MWCNT/SPC	0.086 μg/mL (drop)	2.0 μg/mL	n.s.	[29]
Fentanyl	n.s.	Optical	UV–Visspectrophotometery	RB	0.9 mg/L	n.s.	6 min	[30]
(a) MDA (b) MDMA	n.s.	Electrochemical	DPV	MIP@PDA/Au-E	(a) 37 nM (b) 54 nM	n.s.	n.s.	[31]
Methamphetamine	n.s.	Optical	Fluorescence	CB [7] @BBH	65.2 nM	n.s.	30 s	[32]
Methylamphetamine	n.s.	Electrochemical	DPV	WP5–GDY/GCE	0.016 μM	n.s.	n.s.	[33]
(a) Methamphetamine (b) MDMA	10 μL	Optical	UV–Vis spectrophotometery	1/DMAN impregnatedfilter paper	(a) 0.36 μg/mL (b) 0.57 μg/mL	n.s.	10 min	[34]
Amphetamine	n.s.	Electrochemical	DPV	AMP-Apt/AuNFs@Au	0.51 nM	n.s.	20 min	[35]
Methcathinone	100 μL	Optical	Fluorescence	Eu-MOF	0.40 ng/mL	n.s.	n.s.	[36]
Tramadol	n.s.	Electrochemical	AdASV	(a) H-GONPs/GCE (b) GONSs/GCE	(a) 0.015 μM (b) n.s.	(a) 0.051 μM (b) n.s.	n.s.	[37]
γ-Hydroxybutyric acid (GHB)	n.s.	Optical	UV–Visspectrophotometery	AuNP	n.s.	n.s.	n.s.	[38]

AdSCV: adsorptive-stripping cyclic voltammetry; AuNP: gold nanoparticles; Au/Apt/MCH: Ket-38 aptamer modified Au electrode; AMP-Apt/AuNFs@Au: amphetamine-specific aptamers/gold nanoflowers; Apt-38-MB: aptamer-38-methylene blue; AdASV: adsorptive anodic stripping voltametric; CB[7]@BBH: cationic aggregation-induced emission of berberine hydrochloride with cucurbit[7]uril; Cytc/MWCNT/SPC: cytochrome c/multi-walled carbon nanotubes/screen-printed carbon; CNHs-CHI@PtNPs/GCE: high surface area carbon nanohorns with chitosan acetic acid solution and decorated with Pt nanoparticles/glassy carbon electrodes; Cu/POT/ISM: copper—poly(3-octylthiophene)—ion-sensitive membrane; CSe_2_NF/CC: carbon cloth modified with carbon selenide nanofilms; DPV: differential pulse voltammetry; DMAN: 1,8-bis(dimethylamino)naphthalene; EAB: electrochemical aptamer-based; Eu-MOF: europium metal–organic frameworks; EAB: electrochemical aptamer-based sensor; GONSs/GCE: glassy carbon electrode with graphene oxide nanosheets; H-GONPs/GCE: glassy carbon electrode modified with hierarchical graphene oxide nanoplatelets; LOD: limit of detection; LOQ: limit of quantification; MIP: molecular imprinted polymer; MIP@PDA/Au-E: electrochemical sensor based on molecularly imprinted polydopamine/gold disk electrode; MDA: 3,4-methylenedioxyamphetamine; MDMA: 3,4-methylenedioxymethamphetamine; Nafion-[Ru(bpy)_3_]^2+^: Nafion and tris(2,2′-bipyridyl)-dichlororuthenium(II) hexahydrate; n.s.: not specified; PCFS: portable chemiluminescent fiber-based immunosensor; P-L-Arg/GCE: glassy carbon electrode modified by a thin layer of poly L-arginine; PLS-DA: partial least squares discriminant analysis; POT: polymer poly(3-octylthiophene); WP5–GDY/GCE: water-soluble pillar[5]arene-Graphdiyne/glassy carbon electrode.

**Table 4:** Detection systems for oral fluid.

Compounds	Sample Volume	Type of Sensor	Transducing Mechanism	Sensor Matrix	LOD	LOQ	Time of Analysis	Ref.
Methamphetamine	n.s.	Optical	PCFS	SA-Bio-HRP nanocomposite	0.5 ng/mL	1.5 ng/mL	10 min	[17]
Methamphetamine	n.s.	Electrochemical	EAB	Apt-38-MB	20 nM	n.s.	n.s.	[18]
(a) Methampheta mine (b) Amphetamine (c) *para*-hydroxy- methamphetamine	n.s.	Electrochemical	Electrochemiluminescence	GCE/Nafion/[Ru(bpy)_3_]^2+^	(a) 10 µM (b) 10 µM (c) 10 µM	n.s.	n.s.	[23]
Diazepam	n.s.	Electrochemical	Potentiometry	Cu/POT/ISM	1.2 × 10^−7^ M	n.s.	11 ± 2 s	[25]
Ketamine	n.s.	Electrochemical	EAB	Au/APT/MCH	10 nM	n.s.	30 s	[27]
Tramadol	n.s.	Electrochemical	AdASV	(a) H-GONPs/GCE (b) GONSs/GCE	(a) 0.015 µM (b) n.s.	(a) 0.051 µM (b) n.s.	n.s.	[37]
Methamphetamine	n.s.	Electrochemical	Antibody-antigene recognition	SPGE/IG/mAb	0.72 ng/mL	2.4 ng/mL	n.s.	[39]
(a) Methampheta mine (b) Cocaine	1 mL	Optical	SPR	MA-BSA/COC-BSA/Au SPR chip	(a) 0.95 ng/mL (b) 3.14 ng/mL	n.s.	n.s.	[40]
(a) Cocaine (b) Amphetamine (c) Benzodiazepine	n.s.	Electrochemical	DPV	LSG	(a) 4.3 ng/mL (b) 9.7 ng/mL (c) 9.0 ng/mL	n.s.	n.s.	[41]
(a) Cocaine (b) Heroin (c) MDMA (d) Ketamine	n.s.	Electrochemical	SWAdSV	SDS/Graphite SPE	(a) 1.2 µM (b) 2.4 µM (c) 1.0 µM (d) 2.6 µM	n.s.	n.s.	[42]
Cocaine	n.s.	Optical	SPR	GNP-Aptamer/NaCl	0.97 nM	n.s.	<90 min	[43]
(a) JWH-018 (b) JWH-073 (c) JWH-018 pentanoic acid (d) JWH-073 butanoic acid	n.s.	Gravimetric	Piezoelectric	SCs-MIP QCM	(a) 0.28 pg/mL (b) 0.3 pg/mL (c) 0.23 pg/mL (d) 0.29 pg/mL	(a) 3.03 pg/mL (b) 3.0 pg/mL (c) 2.4 pg/mL (d) 3.1 pg/mL	n.s.	[44]
Pethidine	2 mL	Electrochemical	DPV	CSe_2_NF/CC	19.3 nM	n.s.	n.s.	[45]
25I-NBOMe	n.s.	Optical	Fluorescence	MSNs/Rhodamine B/serotonin derivate/5-HT_2A_	0.6 µM	n.s.	n.s.	[46]
(a) Tetrahydrocannabinol (b) Ethanol	n.s.	(a) Electrochemical (b) Electrochemical	(a) SWV (b) Amperometry	(a) MWCNT/Carbon (b) PB/AOx/Chitosan	(a) 0.5 µM (b) n.s.	n.s.	6 min	[47]

AdASV: adsorptive stripping voltammetry; Apt-38-MB: gold disk electrode with 38-base aptamer sequence labeled with methylene blue; Au/Apt/MCH: gold electrode with aptamers and 6-mercapto-1-hexanol reagent on the surface; CSe2NF/CC: carbon cloth modified with carbon selenide nanofilms; Cu/POT/ISM: copper electrodes modified with poly(3-octylthiophene) and a PVC ion-sensitive membrane; DPV: differential pulse voltammetry; EAB: electrochemical aptamer-based; GCE/Nafion/[Ru(bpy)_3_]^2+^: glassy carbon electrode coated with Nafion and tris (2,2′-bipyridyl)—dichlororuthenium(II) hexahydrate; GNP-Aptamer/NaCl: colloidal gold nanoparticles conjugated to a specific 32-bp aptamer and sodium chloride; GONSs/GCE: glassy carbon electrode modified with graphene oxide nanosheets; H-GONPs/GCE: glassy carbon electrode modified with hierarchical graphene oxide nanoplatelets; LOD: limit of detection; LOQ: limit of quantification; LSG: laser-scribed graphene; MA-BSA/COC-BSA/Au SPR chip: Au SPR chip immobilised with methamphetamine-bovine serum albumin and cocaine-bovine serum albumin; MDMA: 3,4-methylenedioxymethamphetamine; MSNs/Rhodamine B/serotonin derivate/5-HT2A: mesoporous silica nanoparticles loaded with rhodamine B, functionalised with a serotonin derivative and capped with a polyclonal antibody of the 5-HT2A receptor; MWCNT/Carbon: carbon ink modified with a multi-walled carbon nanotube; n.s.: not specified; PB/AOx/Chitosan: carbon electrode modified with carbon-Prussian blue ink, alcohol oxidase, and chitosan; PCFS: portable chemiluminescent fiber-based immunosensor; SA-Bio-HRP nanocomposite: streptavidin-biotin-HRP nanocomposite; SCs-MIP QCM: quartz crystal microbalance chip with synthetic cannabinoid imprinted nanoparticles; SDS/Graphite SPE: unmodified graphite screen-printed electrode enhanced with sodium dodecyl sulfate; SPGE/IG/mAb: screen-printed gold electrode modified with ionogel and a specific monoclonal antibody; SPR: surface plasmon resonance; SWAdSV: square-wave adsorptive stripping voltammetry; SWV: square wave voltammetry.

**Table 5:** Detection systems for sweat.

Compounds	Sample Volume	Type of Sensor	Transducing Mechanism	Sensor Matrix	LOD	LOQ	Time of Analysis	Ref
2-fluoromethampehtamine	n.s.	Optical	SERS	SFF with AgNW	n.s.	n.s.	n.s.	[48]
Cathinone, heroin, cocaine	n.s.	Electrochemical	EAB	(a) Au/APT1/MCH (b) Au/APT2/MCH (c) Au/APT1 + APT2/MCH	(a) 0.32 nM (b) 0.57 nM (c) 0.18 nM	n.s.	n.s.	[49]

LOD: limit of detection; LOQ: limit of quantification; n.s.: not specified; SERS: surface-enhanced Raman scattering; n.a: not available; SFF: silk fibroin protein film; AgNW: plasmonic silver nanowire layer; Au/Apt1/MCH: gold electrode with aptamer 1 and 6-mercapto-1-hexanol reagent on the surface; Au/Apt2/MCH: gold electrode with aptamer 2 and 6-mercapto-1-hexanol reagent on the surface; Au/Apt1 + Apt 2/MCH: gold electrode with aptamers 1 and 2 and 6-mercapto-1-hexanol reagent on the surface.

**Table 6:** Detection systems for hair.

Compounds	Sample Amount	Type of Sensor	Transducing Mechanism	Sensor Matrix	LOD (ng/mL)	Time of Analysis (min)	Ref.
Ketamine	n.s.	Optical	Fluorescence	UC NP’s/LFIA	1	5	[50]
Methamphetamine	5 mg	Optical	Colorimetric Strips	DDNB/LFIA	8	8	[51]
Morphine							

DDNB: deeply dyed nanobeads; LFIA: lateral flow immunoassay; LOD: limit of detection; NP’s: nano-particles; n.s.: not specified; UC: up-converting.

**Table 7:** Type of breathalyser sensors.

Compounds	Sample Volume	Type of Sensor	Transducing Mechanism	Sensor Matrix	LOD	Time of Analysis	Ref.
EtOH	n.s.	Electrical	Amperometry	MQ3/CuO:SnO_2_	230 ng/mL	8 s	[52]
EtOH	n.s.	Electrochemical	Conductometry	SW CNT/PDIC10	0.01% BAC	5 s	[53]
Tetrahydrocannabinol	n.s.	Electrochemical	Conductometry	S-SWCNT	0.163 ng	15 min	[54]
EtOH	1 L	Optical	Colorimetric strips	ALOx/CeNP’s	0.001% (*v*/*v*)	15 min	[55]

ALOx: alcohol oxidase; BAC: blood alcohol concentration; CeNP’s: cerium nano-particles; CNT: carbon nanotubes; ETOH: ethanol; LOD: limit of detection; MQ3/CuO:SnO_2_: metal oxide semiconductor; n.s.: non-specified; PDI: perylene bisimide derivatives; S-SWCNT: semiconductor single-walled carbon nanotubes. SW: single-walled.

**Table 8:** Sensors for the detection of drugs in vapours.

Compounds	Sample Volume	Type of Sensor	Transducing Mechanism	Sensor Matrix	LOD	Time of Analysis (s)	Dissociation Constant (μM)	Ref.
Cocaine Tetrahydrocannabinol Cannabidiol MDMA Heroin Codeine	0.1 ** L/min	Optical	Fluorescence	AgamOBP1_S82P	n.s.	10	0.48 ± 0.14 0.28 ± 0.07 0.06 ± 0.02 0.08 ± 0.01 0.27 ± 0.17 0.65 ± 0.27	[56]
MPEA	n.s.	Optical	Fluorescence	CP S1a/b/tiophene ring structure on UV-ozone treated quartz plates	S1a 2.59 ng/mL/S1d 0.25 ng/mL	150	n.a	[57]
Methamphetamine MDMA Ketamine Magu Phenobarbital	n.s.	Optical	Fluorescence	PBI-CB	5.0 × 10^5^ * 4.0 × 10^5^ * 2.0 × 10^2^ * 2.0 × 10^5^ * 4.0 × 10^4^ *	60	n.a	[58]
NMPEA	n.s.	Electrochemical	Conductometry	CNT functionalised with a polythiophene derivative	4 µg/L	20	n.a	[59]
Ethanol	n.s.	Electrochemical	Conductometry	h-In2O3 NP via the microwave-assisted hydrothermal pathway	330 µg/L	37.6 ± 8.26	n.a	[60]

CNT: carbon nanotubes; LOD: limit of detection; MPEA: methylphenethylamine; n.a: not applicable; NP: nanoparticules; n.s.: non-specified; PBI: perylene bisimide derivatives. * Dilution ratios of air to the equilibrium vapour of the drugs (V/V); ** sample flow rate.

**Table 9:** Overview of electrochemical sensor technologies developed for the detection of illicit drugs in solid samples.

Compounds	Sample Volume	Type of Sensor	Transducing Mechanism	Sensor Matrix	LOD	LOQ	Time of Analysis	Ref.
(a) Methamphetamine (b) MDMA	180 μL	Optical	UV–Vis spectrophotometry	1/DMAN impregnated filter paper	n.s.	n.s.	5 min	[34]
Amphetamine	80 μL	Electrochemical	SWV	SPEs	22.2 μM	n.s.	3 min	[61]
Amphetamine	85 μL	Electrochemical	SWV	PFA-coated SPEs	0.3 mM	0.9 mM	1.5 min	[62]
mCPP	79 μL	Electrochemical	SWV	CPT-BDDE	1.1 μM	3.5 μM	n.s.	[63]
mCPP	100 μL	Electrochemical	SWV	C-SPE	0.1 μM	0.33 μM	n.s.	[64]
LSD	n.s.	Electrochemical	SWV	Paper-based electrodes	0.38 μM	1.27 μM	n.s.	[65]
Fentanyl	n.s.	Electrochemical	SWV	Ag/AgCl	10 µM	n.s	n.s.	[66]
MDEA	5 μL	Electrochemical	SWV	C-SPE	0.03 μM	0.09 μM	n.s.	[67]
Cocaine	n.s.	Electrochemical	SWV	poly(PABA)/GPH-SPE	n.s.	n.s.	7 min	[68]

Ag/AgCl: silver–silver chloride; CPT-BDDE: catodically pre-treated boron-doped diamond electrode; C-SPE: carbon screen-printed electrode; DMAN: 1,8-bis(dimethylamino)naphthalene; LOD: limit of detection; LOQ: limit of quantification; LSD: lysergic acid diethylamide; mCPP: 1-(3-chlorophenyl) piperazine; MDEA: 3,4-methylenedioxyethylamphetamine; MDMA: 3,4-methylene-dioxymethamphetamine; n.s.: non-specified; PFA-coated SPEs: paraformaldehyde-coated graphite screen-printed electrodes; poly(PABA)/GPH-SPE: poly(PABA) electrodeposition-modified graphite screen-printed electrodes; SPEs: screen-printed electrodes; UV–Vis: Ultraviolet–visible; SWV: square wave voltammetry.

## Data Availability

Not applicable.

## Supplementary Materials

The following supporting information can be downloaded at: https://www.mdpi.com/article/10.3390/mi14122249/s1, File S1: Materials and methods - Keywords used in databases.
