# Supplementary material for: Sensors in the Detection of Abused Substances in Forensic Contexts: A Comprehensive Review
Source: Micromachines (Basel). 2023 Dec 17;14(12):2249. doi: 10.3390/mi14122249 (PMC10745465; doi:10.3390/mi14122249)
Supplement: Supplementary file 1 [file micromachines-14-02249-s001.zip › micromachines-2728047-supplementary.pdf]

Supplementary Information: S1: Materials and methods - Keywords used in databases

The search keywords utilized within the PubMed and ISI Web of Science databases encompass the terms "sensors for the detection of drug abuse," "blood derivatives," "urine," "oral fluid or saliva," "sweat," "hair," "breathalyzer sensors," "vapors," "solid samples", “seized samples”, and "powder samples," within all fields. However, with respect to blood and derivatives, urine, and oral fluid samples, only papers published from 2020 to the present were considered for inclusion. For breathalyzer sensors, the search was confined to publications from the last five years. Three authors independently reviewed articles for each category of samples to assess their relevance in the current review; only articles selected by at least two authors were incorporated.
